# Incidence of infections in patients treated with rituximab for autoimmune disorders of hematological Interest or non-Hodgkin lymphoma

**DOI:** 10.1007/s00277-026-07047-4

**Published:** 2026-05-09

**Authors:** Salvatore Perrone, Riccardo Tomasello, Simona Raso, Ombretta Annibali, Mariantonietta Tafuri, Marta Mattana, Tania Soriano, David Fanciullo, Giovanni Manfredi Assanto, Giacinto Luca Pedone, Erminia Baldacci, Eugenio Santacroce, Uros Markovic, Gaetano Giuffrida, Manuela Giuseppa Ingrascì, Mario Biglietto, Claudia Cammarata, Matteo Totaro, Silvio Ligia, Bruno Fattizzo, Cristina Santoro, Sergio Siragusa, Mariasanta Napolitano

**Affiliations:** 1 Hematology, Polo Universitario Pontino, “Sapienza”, Via A. Canova S.M. Goretti Hospital, Latina, Italy; 2 Department of Health Promotion, Mother and Child Care, Internal Medicine and Medical Specialties (ProMISE), University of Palermo, Via del Vespro 129, Palermo, 90127 Italy; 3 Hematology and Rare Diseases Unit, Azienda Ospedaliera Ospedali Riuniti Villa Sofia-Cervello, Palermo, Italy; 4 Division of Hematology, Stem Cell Transplantation, Fondazione Policlinico Universitario Campus Bio Medico, Roma, Italy; 5 Department of precision medicine in medical, surgical and critical care (Me.Pre.Cc), University of Palermo, Raffadali, Italy; 6 Division of Hematology, Department of Translational and Precision Medicine, Azienda Ospedaliera Policlinico Umberto I, Sapienza University of Rome, Rome, Italy; 7 Fondazione IRCCS Ca’ Granda Ospedale Maggiore Policlinico, Milan, Italy; 8 Department of Oncology and Haemato-Oncology, University of Milan, Milan, Italy; 9 Hematology Unit with BMT, A.O.U. Policlinico “G. Rodolico-San Marco”, Catania, 95123 Italy

**Keywords:** Autoimmune hemolytic anemia (AIHA), Immune thrombocytopenia (ITP), Acquired hemophilia A (AHA), Non-hodgkin lymphoma (NHL), Rituximab

## Abstract

Rituximab is employed in the treatment of B-cell non-Hodgkin lymphomas (NHL) and autoimmune disorders of hematological interest (ADHI), such as autoimmune hemolytic anemia (AIHA), acquired hemophilia A (AHA), and immune thrombocytopenia (ITP). Rituximab induces profound B-cell depletion, reducing the titer of immunoglobulins and autoreactive antibodies, but secondary hypogammaglobulinemia increases risk of infections. We performed a retrospective, multicentric study across seven Italian centers to compare two cohorts of patients with either indolent NHL or ADHI treated with rituximab between March 2014 and March 2024. We evaluated the incidence and characteristics of infections and common antimicrobial prophylaxis adopted according to local clinical practice, in the absence of shared guidelines for ADHI. We included 285 patients (52% female; median age 55 years). The ADHI cohort comprised 187 patients (65%; 97 ITP, 78 AIHA, 12 AHA), while the control group included 98 i-NHL patients (34%). We documented 65 infectious events occurring in 65/285 patients (22.8%). Infection-related mortality occurred in 9 patients (4%). When stratified, the cumulative 180-day incidence of infections was 9.4% (IC95% 8.8%-10%) for NHL patients and 13.7% for ADHI (IC95% 12.2%-14.2%). Multivariate analysis identified prophylaxis (OR 2.62, *p* = 0.014) and post-treatment hypogammaglobulinemia (OR 2.73 *p* = 0.004) as risk factors. The risk of infection in patients with ADHI is high and comparable to that observed in NHL. Therefore, close monitoring of IgG levels is strongly advised, and mortality can be contained through rigorous antimicrobial stewardship.

**Supplementary Information:**

The online version contains supplementary material available at 10.1007/s00277-026-07047-4.

## Introduction

Rituximab is a monoclonal antibody that targets CD20, depleting mature B-cells. Since its introduction in 1997 for non-Hodgkin lymphomas (NHL), rituximab has reshaped the treatment of several autoimmune diseases (rheumatoid arthritis, granulomatosis with polyangiitis, and other ANCA-associated vasculitis) [1, 2]. Beyond B-cell depletion, the immunomodulatory effects of rituximab exert decreased autoantibody production, reduced antigen presentation, and disruption of ectopic lymphoid structures [3]. Moreover, some hematological diseases are characterized by autoimmune dysregulation like: Autoimmune Hemolytic Anemia (AIHA) [4], Immune Thrombocytopenia (ITP) [5], and Acquired Hemophilia A (AHA) [6]. Indeed, rituximab is generally used alone or in association with corticosteroids in these conditions, frontline or for relapsed/refractory patients [7–10]. Conversely, rituximab is associated with increased risk of hypogammaglobulinemia, delayed neutropenia, and infections [11, 12]. These complications are particularly severe in patients with NHL treated with rituximab in combination with chemotherapy: in a study in DLBCL patients 39% developed severe infections [13], and in combination with polatuzumab-CHP 13.8% had febrile neutropenia [14]. In indolent NHL receiving rituximab-monotherapy incidence of infections has been reported in fewer cases [15, 16]. Moreover, in patients with autoimmune diseases has been reported a high incidence rate of severe infections after rituximab of 51.0/100 patient-years in systemic lupus erythematosus (SLE) [17] and another study found an incidence of 12.7% in the multiple sclerosis/neuromyelitis optical spectrum disorder group, 27.6% in the ANCA-associated vasculitis group, and 30.6% in the ‘other Auto-immune diseases’ group [18]. On the contrary, there are only limited data on the risk of infections and prophylaxis administration in patients with AIHA, ITP, or AHA. In particular, real-world data on infectious complications and preventive strategies in ADHI patients treated with rituximab remain scarce and heterogeneous.

Therefore, in the present study we decided to assess retrospectively a multicentric cohort of patients with autoimmune disorders of hematological interest (ADHI) and to compare their incidence of infections with a cohort of patients with indolent NHL treated with rituximab single-agent.

## Methods

We collected data regarding infections of patients with a diagnosis of AHA, ITP, AIHA, and indolent NHL (only patients treated with rituximab single-agent were included) treated with rituximab in a real-world setting of seven Italian hematology centers, according to local protocol, at doses of 375 mg/m^2^, between March 2014 and March 2024. The objectives of our study were (a) to assess the incidence and etiology of the infectious complication (b) to assess the mortality due to infections, (c) to describe the prophylactic approaches used, given the current absence of clear guidelines or recommendations for ADHI [19, 20]. Data on concomitant corticosteroid use were not systematically available across centers and were therefore not included in the analysis.

Baseline and post-rituximab immunoglobulin (Ig) plasma levels were assessed according to local clinical practice; hypogammaglobulinemia was defined as IgG levels < 500 mg/dL measured after rituximab exposure; Ig administration (0.4 g/kg IV) for hypogammaglobulinemia was given according to practice of each treating center [21].

All documented bacterial infections requiring intravenous treatment (classified as CTCAE.5 grade three or higher) and invasive fungal infection (IFI) classified as proven, probable and possible, according to the European Organization for Research and Treatment of Cancer (EORTC) and the Mycoses Study Group Education and Research Consortium (MSG) definitions were included [22]. Febrile events without microbial identification, defined as fever of unknown origin (FUO), were separately analyzed. Bacteremia by coagulase-negative staphylococci (CoNS) and by Corynebacteria was considered only when supported by at least two positive blood cultures. We excluded non‐IFI and the cases of colonization by multidrug‐resistant bacteria. Recurrent infections caused by the identical etiologic agent and arising in the same patient were considered as one event. The incidence/100 patients/year was calculated dividing the total number of infections for the time of exposition of patients.

We collected data about the prophylactic treatments administered according to the policy of each center. The initiation and duration of antimicrobial prophylaxis were not standardized across centers and were based on individual clinical judgment. Prophylaxis was generally initiated concomitantly with the first exposure to rituximab. Given the observational design, antimicrobial prophylaxis was treated as a fixed baseline variable and may reflect underlying patient risk; therefore, potential confounding by indication was anticipated and considered in the interpretation of regression analyses. The incidence of *Pneumocystis jirovecii* pneumonia (PJP) and the impact of specific antimicrobial prophylaxis were analyzed separately from fungal events due to the different prophylaxis administered.

A statistical analysis was conducted, where the continuous variables were expressed as median and interquartile range (IQR) and compared using the Kruskal–Wallis test, due to non-normal distribution. Categorical variables were represented as absolute numbers and percentages, and these were analyzed using the Pearson chi-square test. Univariate logistic regression analyses were conducted to evaluate the association between demographic and clinical variables and the presence of infection. Variables that were deemed to be clinically relevant, or that demonstrated an association in univariate analyses, were included in multivariate logistic regression models in order to identify independent predictors. The results were reported as odds ratios (ORs) with 95% confidence intervals (95% CI), with a p value of < 0.05 being considered to be statistically significant. Statistical analysis was conducted with Jamovi ver. 2.6.44, using Clinicopath modules [23]. Incidence of infections was calculated with 1-Kaplan-Meyer curves in SPSS, ver. 26.

The study was conducted in accordance with the Declaration of Helsinki and approved by the Ethics Committee of the coordinating center (University Hospital “P. Giaccone”). Given its retrospective design, written informed consent was waived where allowed by local regulations; nevertheless, written informed consent was obtained from patients whenever feasible. Data were collected and analyzed in anonymized form.

## Results

**Fig. 1:**
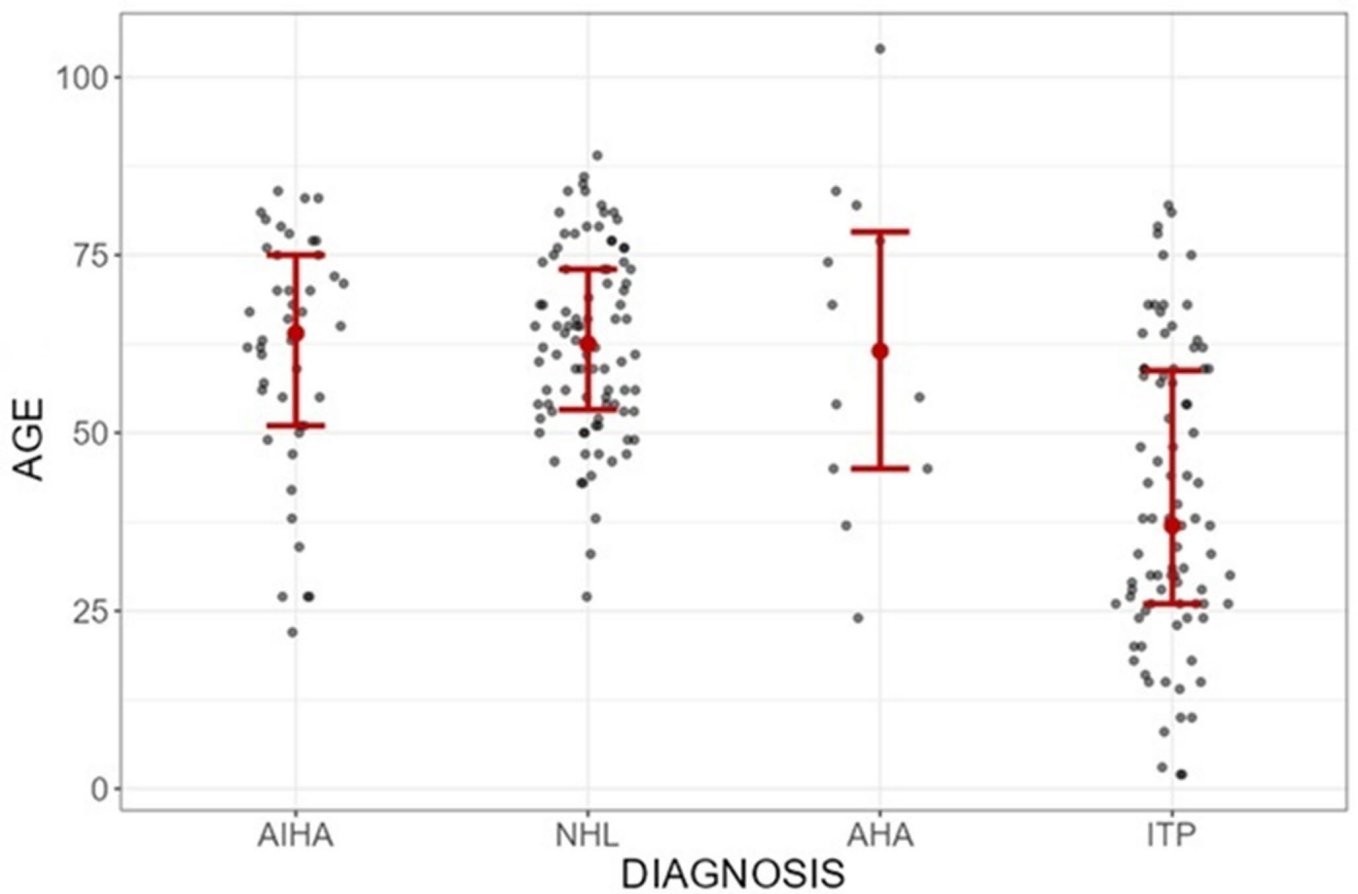
Age distribution at diagnosis across clinical cohorts (Jittered scatter plot). Individual data points (grey circles) represent patient age (in years) for the four diagnostic groups: Autoimmune Hemolytic Anemia (AIHA), Non-Hodgkin Lymphoma (NHL), Acquired Hemophilia A (AHA), and Immune Thrombocytopenia (ITP). Red central markers indicate the mean age for each group, with error bars representing the standard deviation (±SD)

The main baseline characteristics of the 285 patients are summarized in Table 1. Of these, 148 were female (52%) and 137 males (48%); the median age at diagnosis was 56 years (Q1-Q3: 38–68 years), while that at first treatment with rituximab was 59 years (Q1-Q3: 46–73 years). We identified 187 (65%) patients with autoimmune disorders of hematological interest (ADHI); of these, 97 had ITP (34%), 78 had Autoimmune Hemolytic Anemia (AIHA) (27%), and 12 patients acquired Hemophilia A (AHA) (4.2%). The control group was represented by 98 patients (34%) with indolent-NHL. The age distribution for diagnostic category is represented in Fig. 1 and shows that patients with ITP were younger (median 42 years), while the median age for the group of i-NHL, AHA, and AIHA was 62 years (*P* < 0.001).

**Table 1:** Demographic and clinical features of study cohort

Total number of Patients	285
SEX	
MALE (1)	137 (48)
FEMALE (0)	148 (52)
AGE AT DIAGNOSIS (years)	MEDIAN (Q1;Q3)
	56 (38;68)
AGE AT RITUXIMAB (years)	MEDIAN (Q1;Q3)
	59 (46;73)
DIAGNOSIS	
NHL	98 (34)
ADHI	187 (65)
AIHA	78 (27)
AHA	12 (4.2)
ITP	97 (34)
PROPHYLAXIS	
PJP (1)	138 (49)
NO PJP (0)	147 (51)
DURATION OF PROPHYLAXIS (MONTHS)	MEDIAN (Q1;Q3)
	8 (6;12)
GAMMAGLOBULINEMIA AT THE END OF RITUXIMAB	
NORMAL OR ELEVATED	230 (80)
REDUCED	55 (20)
INFECTIOUS COMPLICATIONS	
YES	65 (22.8)
NO	220 (77.2)
BY DIAGNOSIS	
AHA	3 (25%)
AIHA	21 (27%)
ITP	23 (24%)
NHL	18 (18%)
SITE OF INFECTION	
AIRWAYS	32 (11.2)
BLOODSTREAM INFECTIONS (BSIs)	11 (4)
GENITOURINARY	10 (3.5)
CUTANEOUS	6 (2)
GASTROINTESTINAL	6 (2)
GRADE OF INFECTION (CTCAE)	
I	13 (4.5)
II	30 (10)
III	12 (4.2)
IV	9 (3)
* Not available*	1
GRADE III/IV BY DIAGNOSIS	
AHA	1 (1)
AIHA	5 (1)
ITP	8 (3)
NHL	7 (1)
STATUS AT END OF OBSERVATION	
ALIVE	242 (85)
DECEASED/LOST AT FU	43 (15)
CAUSE OF DEATH	
RELATED TO INFECTION	9 (3.1)
UNRELATED TO INFECTION/LOST AT FU	34 (12)

*Name of variable (representative value); number (%)*,* unless stated otherwise*

We documented 65 infectious events in 22% of patients. The overall incidence rate was 14.7 infections per 100 patient-years. Overall, there were 60/65 (92.3%) bacterial events, 1/65 (1.5%) fungal, 3/65 (4.6%) viral, and 1/65 (1.5%) PJP events. According to CTCAE v5.0, infections were graded as follows: 13 Grade I, 30 Grade II, 12 Grade III, and 10 Grade IV events (total *n* = 65). (Supplemental Table 1). The mortality was correlated to infections in 9 patients (4%). The infection site was stratified in Table 2. In more detail, we found 32 airways infections (49%), 11 Bloodstream infections (BSIs) (17%), 10 urinary-tract infections (UTI) (15%), 6 skin infections (9%), and 6 gastro-intestinal (9%).

**Table 2:** Patients stratified by site of infection

Cross Table for Dependent INFECTION_SITE							
	*N*	Airways	G.I.	SEPSIS/BSI	SKIN	UTI	Test Statistic
		(*N* = 32)	(*N* = 6)	(*N* = 11)	(*N* = 6)	(*N* = 10)	
AGE_at treatment	285	55.2 64.5 72.7	41.2 56.5 61.2	55.0 73.0 77.7	22.3 53.0 71.3	57.6 74.5 76.1	F_4,60_=1.63, *P* = 0.18^1^
SEX	285	0.5 17/32	0.5 3/6	0.6 7/11	0.5 3/6	0.2 2/10	Χ_24_ = 4.53, *P* = 0.34^2^
DIAGNOSIS	285						Χ^2^_12_ = 7.72, *P* = 0.81^2^
AHA		0.0 1/32	0.0 0/6	0.1 1/11	0.0 0/6	0.1 1/10	
AIHA		0.3 10/32	0.5 3/6	0.3 3/11	0.5 3/6	0.2 2/10	
ITP		0.3 10/32	0.2 1/6	0.4 4/11	0.5 3/6	0.5 5/10	
NHL		0.3 11/32	0.3 2/6	0.3 3/11	0.0 0/6	0.2 2/10	
PROPHYLAXIS	285	0.9 28/32	0.7 4/6	0.7 8/11	0.7 4/6	0.8 8/10	Χ^2^_4_ = 2.82, *P* = 0.59^2^
Hypogamma	285	0.4 12/32	0.5 3/6	0.5 6/11	0.5 3/6	0.2 2/ 10	Χ^2^_4_ = 3.22 , *P* = 0.52^2^

N is the number of non-missing value. ^1^Kruskal-Wallis. ^2^Pearson. ^3^Wilcoxon

G.I. gastro-intestinal. BSI Bloodstream infection. UTI Urinary-tract infections. AIHA autoimmune hemolytic anemia, AHA acquired hemophilia A, and ITP immune thrombocytopenia. NHL Non-Hodgkin Lymphoma

**Fig. 2:**
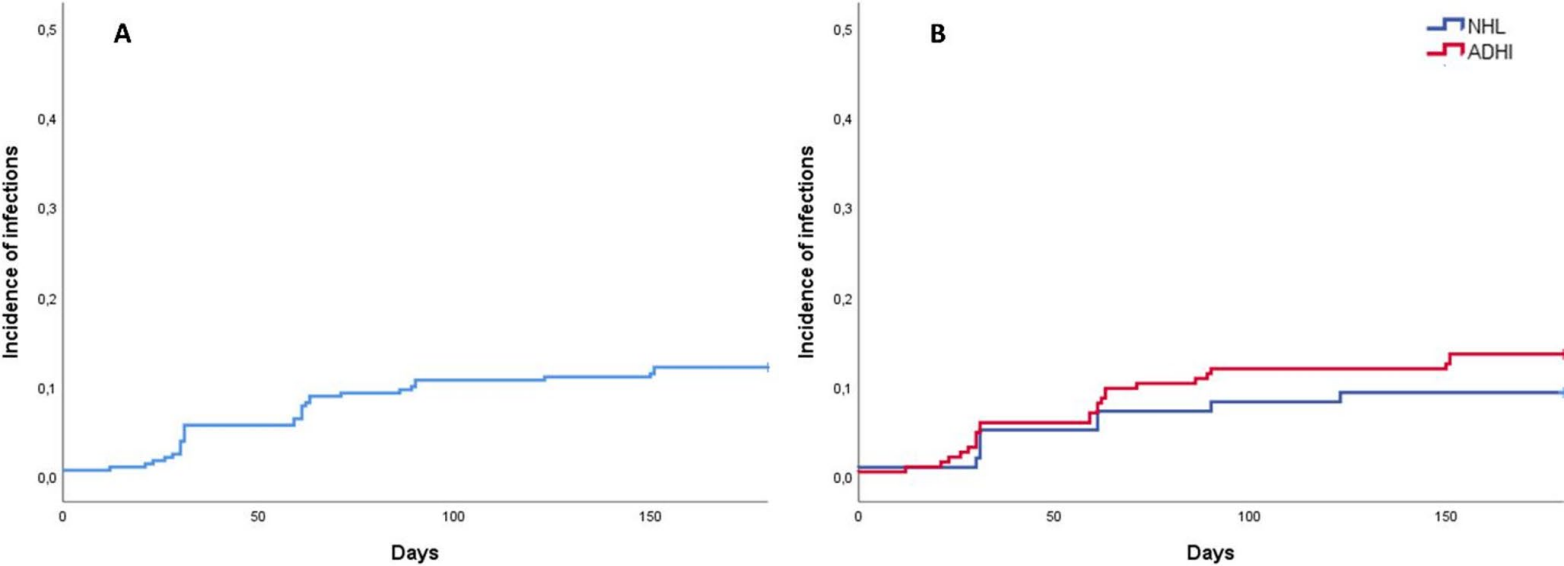
**A**. The cumulative incidence of infections in the whole population at 180-day. The cumulative incidence was 12.2% (IC95% 8.28%-16.12%) **B**. The cumulative 180-day incidence of infections for NHL (9.4%) patients and ADHI (13.7%), not statistically significant difference (*P*=0.2)

The cumulative incidence of infections in the whole population at 180-day was 12.2% (IC95% 8.28%-16.12%). When stratified, the cumulative 180-day incidence of infections was 9.4% (IC95% 8.8%-10%) for NHL patients and 13.7% for ADHI (IC95% 12.2%-14.2%). This difference was not statistically significant (*P* = 0.20). (Fig. 2).

Furthermore, we compared patients who developed infections and those patients who did not (*N* = 220) (Table 3). Patients who developed infections were older, with a median of 65 years, vs. 59 years for those who did not (*P* = 0.19). Infections occurred in 20 out of 55 patients with post-rituximab hypogammaglobulinemia (36%) versus 28 out of 230 patients without hypogammaglobulinemia (16%) (*P* < 0.001).

**Table 3:** Patients stratified by occurrence of infection

Cross Table for Dependent INFECTION				
	*N*	No infection	Yes infections	Test Statistic
		(*N* = 219)	(*N* = 65)	
AGE_R	285	47.0 **59.0** 72.0	52.0 **65.0** 75.0	F_1,282_=1.76, *P* = 0.19^3^
SEX : 1	285	0.5 104/219	0.5 32/65	Χ^2^_1_ = 0.06, *P* = 0.80^2^
DIAGNOSIS	285			Χ^2^_3_ = 1.82, *P* = 0.61^2^
AHA		0.0 9/219	0.0 3/65	
AIHA		0.3 57/219	0.3 21/65	
ITP		0.3 74/219	0.4 23/65	
NHL		0.4 79/219	0.3 18/65	
PROPHYLAXIS	285	0.7 143/219	0.8 52/65	Χ^2^_1_ = 5.04, *P* = 0.02^2^
Hypogamma	285	0.2 38/223	0.4 26/62	Χ^2^_1_ = 17.27, *P* < 0.01^2^

N is the number of non-missing value. ^1^Kruskal-Wallis. ^2^Pearson. ^3^Wilcoxon

AIHA autoimmune hemolytic anemia, AHA acquired hemophilia A, and ITP immune thrombocytopenia. NHL Non-Hodgkin Lymphoma

We collected data on anti-infection prophylaxis administered to 201 patients (70%). In detail, 192 patients received anti PJP prophylaxis with trimethoprim/sulfamethoxazole (TMP/SMX), of these 132 in association with anti-viral prophylaxis with acyclovir [for herpesvirus reactivation: particularly herpes simplex virus (HSV) and varicella zoster virus (VZV)]. Only 7 patients received acyclovir as single agent, and 9 patients received an unknown type of prophylaxis (missing data). For HBV reactivation prophylaxis, 7 patients received lamivudine and 2 patients entecavir (Table 4). Of the 65 patients who developed infections, 52 had received a prophylaxis (80%), while of the 220 patients who did not develop infections, 143 had received prophylaxis (65%) (*P* = 0.01). However, the group of patients that received prophylaxis was different from the group of patients that did not have prophylaxis (Supplemental Table 2). In particular, 20% of patients older than 70 years did not receive prophylaxis, while 34% of patients older than 70 years old received prophylaxis (*P* = 0.02); of the 96 patients with NHL 91, only 7 did not receive prophylaxis, and of the 97 patients with ITP, 51% did not receive prophylaxis (Supplemental Table 1).

**Table 4:** Type of prophylaxis and occurrence (or not) of infection

Cross Table for Dependent INFECTION				
	*N*	No infection	Infection	Test Statistic
		(*N* = 219)	(*N* = 65)	
PROPHYLAXIS_DETAIL	201			Χ^2^_5_ = 7.20, *P* = 0.21^2^
TMP/SMX		0.3 42/145	0.2 12/55	
TMP/SMX + Acyclovir		0.6 92/145	0.7 38/55	
TMP/SMX +Acyclovir + Entecavir		0.0 1/145	0.0 1/55	
Acyclovir		0.0 4/145	0.1 3/55	
TMP/SMX + Acyclovir + Lamivudine		0.0 6/145	0.0 0/55	
Lamivudine		0.0 0/145	0.0 1/55	

N is the number of non-missing value. ^1^Kruskal-Wallis. ^2^Pearson. ^3^Wilcoxon. TMP/SMX=trimethoprim-sulfamethoxazole

**Fig. 3:**
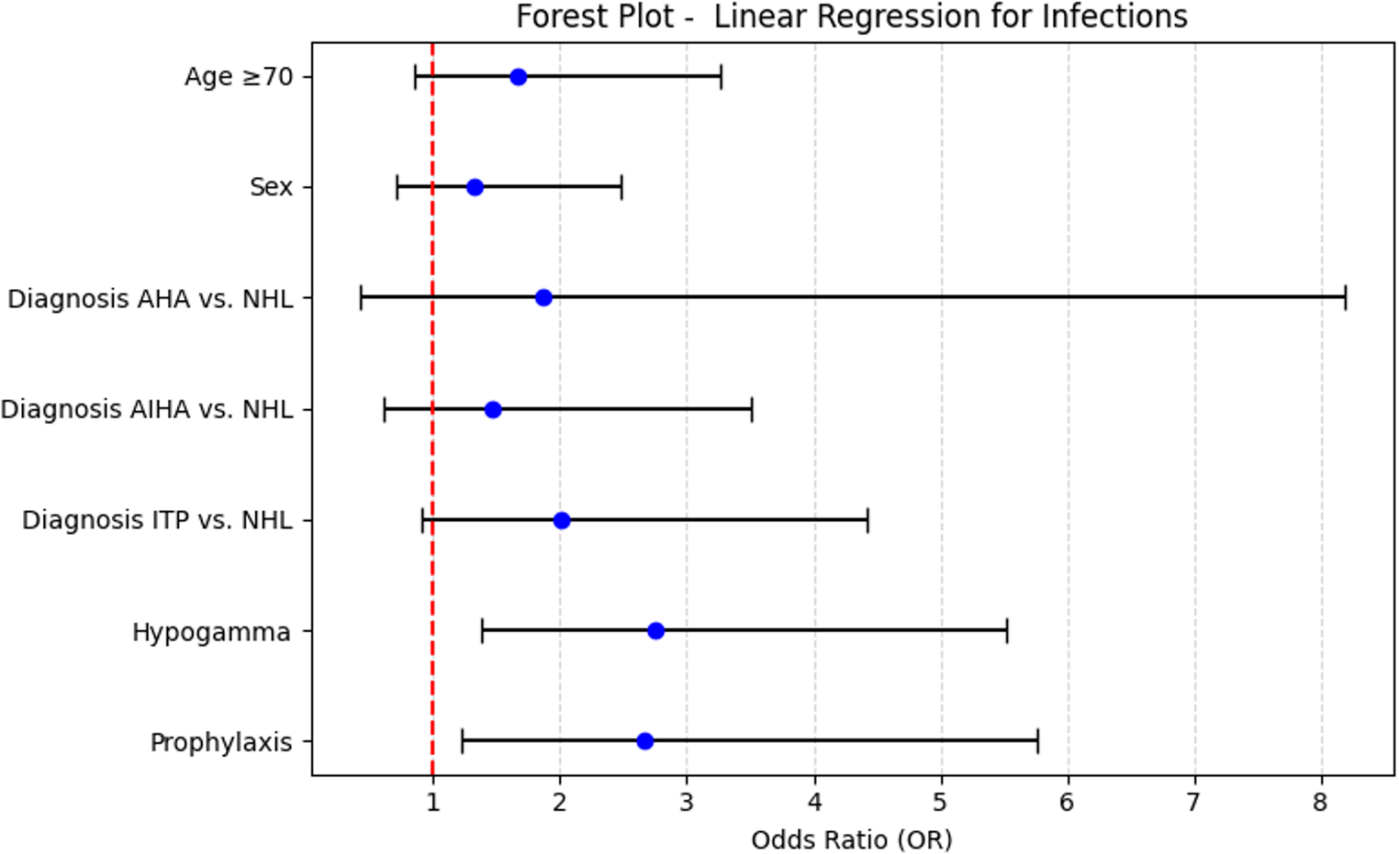
Forest-plot of Odds Ratio (OR) of the risk factors associated with infections

We studied risk factors associated with the onset of infection in univariate and logistic-regression (multivariate analysis) for age > 70 years; gender; type of diagnosis sub-group; use of prophylaxis, and, hypogammaglobulinemia. In univariate analysis (Supplemental Table 3) we found a statistically significant association of infection with the absence of prophylaxis (OR 2.126, IC95% 1.09–4.15, *p* = 0.027), and hypogammaglobulinemia (OR 2.807 IC95% 1.45–5.41, *p* = 0.002). In multivariate analysis, antimicrobial prophylaxis was associated with increased infection risk (OR 2.62, 95% CI 1.21–5.68; *p* = 0.014); however, this likely reflects confounding by indication, as patients receiving prophylaxis were older and had higher baseline clinical risk profiles (see Fig. 3, for forest plot of OR and Supplemental Table 2).

## Discussion

Rituximab can be considered an “old drug” in the field of hematological malignancies, and its profile of safety is well managed by clinicians treating NHL. Nevertheless, its safety profile in non-neoplastic hematological diseases is less studied, particularly regarding the frequency and type of infections, and the need for primary anti-infectious prophylaxis. In this large multicentric cohort, the cumulative incidence of infections was 22%, and it was consistent among all diagnostic categories.

Prior studies showed that ITP patients have a non-negligible incidence of infections after rituximab: in RCT, the rate of infections was 40% [24], in a registry study was 2.3 infections/100 patient-years [25], and in our previous experience it was 15% [26]. Regarding AIHA, data are more limited. Indeed, a study that included several autoimmune cytopenias treated with rituximab found the 1- and 2-year incidence of severe infections of 17.3 and 11.3 per 100 person-years, respectively and they were mostly bacterial pneumonias (45%) and bacteremias (21%) [27]. A recent large French study collected 959 AIHA patients treated with rituximab where the 6-month cumulative incidence of hospitalization with infection was 17.6% [28]. In a real-world study conducted in the USA on 611 patients with cold AIHA, 48.8% had at least one episode of severe infections after rituximab use [29]. Infections were mainly associated with age older than 70 years and with concomitant use of high dose steroids.

We here compared the incidence of infections and found it was similar between ADHI and iNHL. This is a remarkable result because autoimmune and neoplastic disease, with different etiologies, fared similarly after rituximab.

Infection-related mortality occurred in 9 patients (4%). In a Spanish registry study in AHA the incidence of mortality from infections was high, 9.9% [30], and 9% in a Finnish study [31].

There are not specific guidelines on prophylaxis in patients with ADHI, although prophylaxis against *Pneumocystis jirovecii* pneumonia (PJP) with trimethoprim-sulfamethoxazole (TMP/SMX) is recommended for patients receiving rituximab who have additional risk factors, such as concomitant high-dose glucocorticoids (≥ 30 mg/day prednisone equivalent for ≥ 4 weeks) or underlying hematologic malignancy. This approach significantly reduces PJP incidence and is supported by favorable risk-benefit analyses [19, 20, 32]. In our study, the 7 centers adopted their own practice on TMP/SMX prophylaxis and Ig supplementation, resulting in a lack of homogeneity, and a single case of PJP was documented, the patient was not on prophylaxis. Notably, severe infections (CTCAE grade 3–5) accounted for a substantial proportion of events across the entire cohort and largely occurred during prolonged follow-up, underscoring the clinical relevance of the observed infection-related mortality.

In the current study, patients exposed to anti-PJP and anti-herpetic virus prophylaxis had a higher incidence of infections. This finding should not be interpreted as a harmful effect of prophylaxis but rather as a possible marker of higher baseline infectious risk. Another possibility is that it indicates a “confounding by indication”, meaning clinicians were correctly identifying the highest-risk population to give them prophylaxis, but this still led to infections probably because the drugs used (e.g., TMP/SMX) did not cover bacteria involved in BSI and pneumonia. Indeed, patients who received prophylaxis were older and almost all patients with NHL received prophylaxis, while about half patients with AIDH did, supporting the interpretation of prophylaxis as a marker of higher clinical frailty rather than a causal risk factor. It is challenging to identify antibacterial drugs for prophylaxis since development of antibiotic resistance represents a major concern and in neutropenic patients, fluoroquinolones have limited effects in reducing mortality, and there is a worldwide increase in multi-drug resistant strains [33]. However, prophylaxis with TMP/SMP was very effective since we found only one PJP events in our dataset, differently from the 11 cases of pneumocystosis (1%) reported by Zadro et al. [28]. Indeed, the vast majority of patients received TMP/SMX that significantly reduced the overall PJP incidence in patients with rheumatic diseases receiving rituximab treatment [20]. Moreover, in patients who received HBV anti-viral prophylaxis we did not have HBV re-activation.

Hypogammaglobulinemia was the main risk factors for infections identified in patients with ADHI receiving rituximab. However, the retrospective design did not allow modeling hypogammaglobulinemia as a time-dependent covariate, which may have led to residual confounding. In any case, these findings suggest the opportunity of monitoring IgG levels in these patients [21, 34] and to consider IVIG supplementation in those with prolonged hypogammaglobulinemia and repeated infections. Advanced age did also predispose to infections, consistently with other previous reports [28].

Given the retrospective multicenter design and the primarily descriptive aim of the study, no propensity score adjustment was performed; however, this may have resulted in residual confounding. We had no data on vaccination practice adopted by the different centers, that can have beneficial effects also in patients with ADHI [35, 36]. We did not collect systematic data on concomitant corticosteroid use, a relevant modifier of infectious risk, which may have contributed to residual confounding.

In conclusion, we report similar real-world incidence of infections in patients treated with rituximab for ITP, AIHA, and AHA compared to patients with iNHL. Infections were associated with hypogammaglobulinemia suggesting a role for IVIG prophylaxis in selected cases. Anti-infectious prophylaxis was not fully protective regarding the risk of infections, with the exception of cotrimoxazole use for PJP prevention. Further research is warranted to elucidate these effects in the evolving scenario of increased multi-drug resistance and increased infectious complications in NHL patients receiving CD3/CD20 bispecific antibodies [37, 38], since they could also have a role in autoimmune diseases [39] in the next future.

## Supplementary Information

Below is the link to the electronic supplementary material.

Supplementary Material 1 (DOCX 109 KB)

## Data Availability

The data that support the findings of this study are available from the corresponding authors upon reasonable request.
