# Supplementary material for: Incidence of infections in patients treated with rituximab for autoimmune disorders of hematological Interest or non-Hodgkin lymphoma
Source: Ann Hematol. 2026 May 9;105(7):303. doi: 10.1007/s00277-026-07047-4 (PMC13309433; doi:10.1007/s00277-026-07047-4)

**Incidence of infections in patients treated with rituximab for Autoimmune Disorders of Hematological Interest or Non-Hodgkin Lymphoma**

**Supplemental Material**

**Supplemental Table 1**. The Site of infection is stratified according to CTCAE 5.0 grading. **
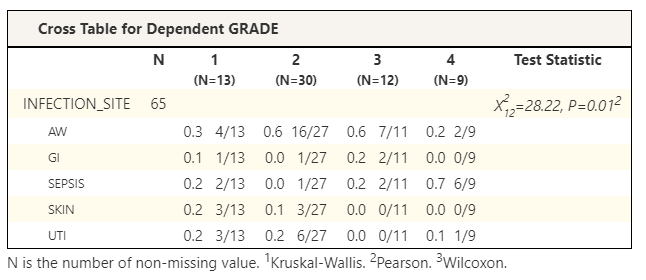
**

**Supplemental Table 2**. Clinical differences in patient who not received (0 column) ore received (1 column) anti PCP Prophylaxis.

**
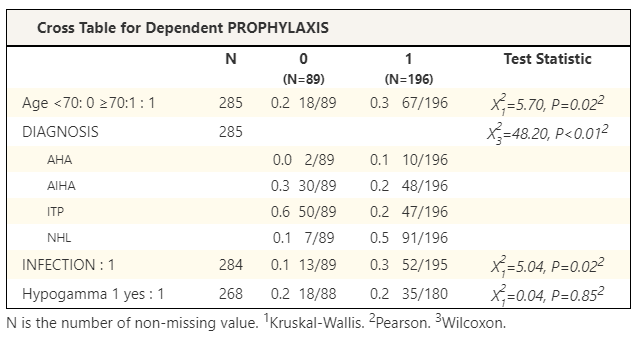
**

**Cross Table for Dependent PROPHYLAXIS**

|  | **N** | **No proph.** | **Yes prophylaxis** | **Test Statistic** |
| --- | --- | --- | --- | --- |
|  |  | (N=89) | (N=196) |  |
| **Age <70: 0 ≥70:1 : 1** | 285 | 0.2  18/89 | 0.3   67/196 | Χ^2^_1_=5.70, P=0.02^2^ |
| **DIAGNOSIS** | 285 |  |  | Χ^2^_3_=48.20, P<0.01^2^ |
| **AHA** |  | 0.0   2/89 | 0.1   10/196 |  |
| **AIHA** |  | 0.3  30/89 | 0.2   48/196 |  |
| **ITP** |  | 0.6  50/89 | 0.2   47/196 |  |
| **NHL** |  | 0.1   7/89 | 0.5   91/196 |  |
| **INFECTION** | 284 | 0.1  13/89 | 0.3   52/195 | Χ^2^_1_=5.04, P=0.02^2^ |
| **Hypogamma** | 268 | 0.2  18/88 | 0.2   35/180 | Χ^2^_1_=0.04, P=0.85^2^ |

**N is the number of non-missing value. ^1^Kruskal-Wallis. ^2^Pearson. ^3^Wilcoxon.**

**Supplemental Table 3** Logistic regression showing Odd Ratios (OR) in univariable and multiviable analysis.

| Risk-factors |  | OR (univariable) | OR (multivariable) |
| --- | --- | --- | --- |
| Age <=70=0; >70=1 |  |  |  |
|  | 1 | 1.655 (1.94-3.03, p=0.076) | 1.53 (0.78-2.98, p=0.208) |
| SEX | 0 | - | - |
|  | 1 | 1.07 (0.62-1.87, p=0.805) | 1.35 (0.72-2.53, p=0.347) |
| DIAGNOSIS | AHA | - | - |
|  | AIHA | 1.10 (0.27-4.48, p=0.889) | 0.78 (0.17-3.58, p=0.752) |
|  | ITP | 0.93 (0.23-3.74, p=0.921) | 1.04 (0.23-4.39, p=0.996) |
|  | NHL | 0.68 (0.17-2.78, p=0.595) | 0.49 (0.11-2.11, p=0.333) |
| PROPHYLAXIS |  | - | - |
|  |  | 2.126 (1.09-4.15, p=0.027) | 2.62 (1.21-5.67, p=0.014) |
| Hypo-GAMMA |  | - | - |
|  |  | 2.807 (1.45-5.41, p=0.002) | 2.73 (1.37-5.45, p=0.004) |

**Supplemental figure** showing histograms of age distribution of the 4 cohorts of patients: AHA acquired hemophilia A; AIHA autoimmune hemolytic anemia; ITP immune thrombocytopenia; NHL B-cell non-Hodgkin lymphomas.


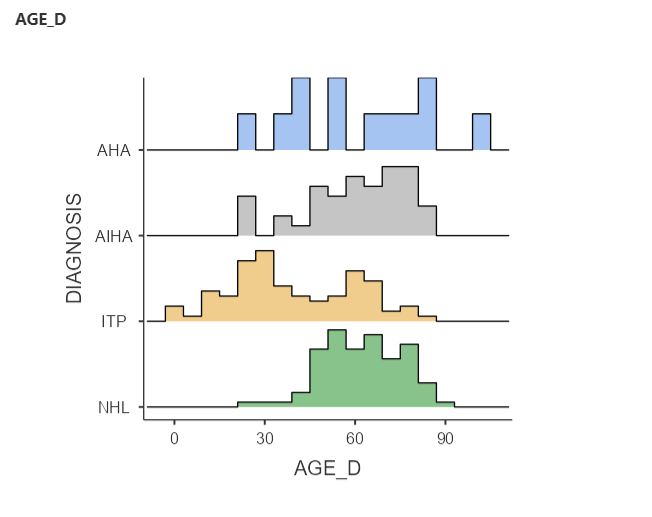

Supplement: Supplementary file 1 — Supplementary Material 1 (DOCX 109 KB) [file 277_2026_7047_MOESM1_ESM.docx]
